# Iron Is Filtered by the Kidney and Is Reabsorbed by the Proximal Tubule

**DOI:** 10.3389/fphys.2021.740716

**Published:** 2021-09-30

**Authors:** Mark Wareing, Craig P. Smith

**Affiliations:** School of Medical Sciences, The University of Manchester, Manchester, United Kingdom

**Keywords:** kidney, iron, micropuncture, proximal tubule, glomerular filtration, DMT1, ZIP8, ZIP14

## Abstract

The aim of this study was to determine the iron (Fe) concentration profile within the lumen of the S2 renal proximal convoluted tubule (PCT) and to resolve whether this nephron segment transported Fe. To do this, we performed *in vivo* renal micropuncture on Wistar rats, collected PCT tubular fluid from superficial nephrons, and measured Fe concentration. The Fe concentration profile along the S2 PCT suggested significant Fe reabsorption. Proximal tubules were also microperfused *in vivo* with physiological solutions containing Fe and Zn, Cu, Mn, or Cd. PCTs perfused with 12μmol.l^−1 55^FeCl_3_ reabsorbed 105.2±12.7 fmol.mm^−1^.min^−1^ Fe, 435±52pmol.mm^-1.^min^−1^ Na, and 2.7±0.2nl.mm^−1^.min^−1^ water (mean ± SEM; *n*=19). Addition of ascorbate (1mmol.l^−1^) to the perfusate did not significantly alter Fe, Na, or water reabsorption. Supplementing the control perfusate with 60μmol.l^−1^ FeSO_4_ significantly decreased ^55^Fe uptake. Recalculating for the altered molar activity following addition of unlabeled Fe revealed a three-fold increase in Fe flux. Addition to the perfusate 12μmol.l^−1^ CuSO_4_, MnSO_4,_ CdSO_4,_ or ZnSO_4_ did not affect Fe, Na, or water flux. In conclusion, (1) *in vivo*, S2 PCTs of rat reabsorb Fe and (2) Fe is reabsorbed along the PCT *via* a pathway that is insensitive to Cu, Mn, Cd, or Zn. Together, these data demonstrate for the first time the hitherto speculated process of renal Fe filtration and subsequent tubular Fe reabsorption in a living mammal.

## Introduction

Iron (Fe) is an essential metal for life. Evolution has harnessed its favorable redox potential, and consequently, Fe is a key constituent of numerous essential proteins, including hemoglobin, cytochromes, and NADH-coenzyme Q reductase (Ponka, 1999; Williams, 2012). Organisms have evolved mechanisms that exercise fine control over Fe balance because Fe deficiency or excess result in morbidity and mortality. The discovery of Fe transporter proteins expressed in kidney nephron epithelial cells has led to the suggestion that Fe is reabsorbed by the kidney (Gunshin et al., 1997; Kozyraki et al., 2001; Wolff et al., 2011; Smith et al., 2019). Yet, a key piece of knowledge is lacking in support of this suggestion, namely, there are no direct measures of Fe in kidney tubular fluid or ultrafiltrate. Not surprisingly, in the absence of this fundamental piece of information, the prevailing dogma persists that healthy kidneys do not filter Fe because Fe is tightly bound to transferrin and in the absence of pathology transferrin is not filtered.

Since the discovery and characterization of the first mammalian Fe transporting protein DMT1 (Gunshin et al., 1997), we have worked to determine the role of this and other Fe transporting proteins in the kidney (Smith and Thévenod, 2009; Thévenod and Wolff, 2016). From our work and that of others, it is well established that the components necessary for transcellular Fe transport are expressed in mammalian proximal convoluted tubule (PCT) cells. Briefly, the membrane-bound divalent metal transporters DMT1, ZIP8, ZIP14, and ferroportin 1 are expressed in PCT cellular membranes (Abouhamed et al., 2006; Wolff et al., 2011; Ajjimaporn et al., 2012; Van Raaij et al., 2018). Furthermore, transferrin receptor 1 and the megalin/cubilin receptor complex, both suggested to mediate reabsorption of filtered protein-bound Fe, are expressed in the apical PCT membrane (Christensen et al., 2012; Smith et al., 2019).

Despite this relative wealth of knowledge, the key questions whether Fe is present in ultrafiltrate and if so whether Fe is reabsorbed by the kidney remain unanswered. In view of these gaps in our collective knowledge, the aim of this study was to determine the Fe concentration in PCT S2 rat ultrafiltrate, determine whether the proximal tubule reabsorbs Fe, and begin to characterize the pathway. To do this, we collected nanoliter samples of PCT fluid from anesthetized rats and measured Fe concentration in tubular fluid along the PCT. In addition, using *in vivo* microperfusion, we measured PCT unidirection flux of radiolabeled Fe (^55^Fe) and assessed the effect of competition by cadmium, copper, manganese, or zinc on ^55^Fe flux.

## Materials and Methods

### Study Approval

All animal studies were performed in accordance with the United Kingdom Animals (Scientific Procedures) Act 1986 and were approved by the University of Manchester Ethics Committee.

### Experimental Procedure

Micropuncture experiments were performed on male Wistar rats (253±8g; *n*=30). Anesthesia was induced with Inactin (5-ethyl-5(1^′^-methyl-propyl)-2-thiobarbiturate; RBI) at a dose of 100mgkg^−1^ i.p. Once a satisfactory level of anesthesia was achieved (assessed by the absence of pinch and corneal reflexes), the animal was placed on a thermostatically controlled table set to maintain body temperature at 37°C. The animal was prepared for micropuncture as previously described (Green et al., 1974). Upon completion of surgery, animals with a proximal tubule transit time in excess of 12s or a mean arterial pressure (MAP) below 100mmHg were rejected from further analysis.

### Collection of Tubular Fluid From Proximal Convoluted Tubules Using an Oil Blockade

Individual PCTs were punctured at random with an oil-filled glass micropipette. An oil block was injected into the tubule lumen, and a timed collection of luminal fluid was made. Two to five collections were made per animal. At the end of an experiment, arterial blood (5-10ml) was withdrawn *via* the descending aorta and the animals were subsequently given an overdose of anesthetic. To determine the distance of the collection site from the glomerulus, the collection sites were identified, the tubule lumen was filled with a silicone rubber solution (Microfil; Flow Tek, Boulder, CO), and the filled kidney was removed and stored overnight in deionized water at 4°C. Casts were then microdissected and measured as previously described (Green et al., 1974).

### Microperfusion of Proximal Convoluted Tubules

Proximal convoluted tubules were perfused at 25nl.min^−1^ with physiological solutions using a continuous microperfusion technique (see Bank and Aynedjian, 1972). The control perfusate contained 12μmol.l^−1^ FeCl_3_, 140mmol.l^−1^ NaCl, 5mmol.l^−1^ NaHCO_3_, 3.5mmol.l^−1^ KCl, 1mmol.l^−1^ CaCl_2_, 0.5mmol.l^−1^ MgCl_2,_ 0.05% erioglaucine dye, ^14^C-inulin at 12.5μCi.ml^−1^ (specific activity=4.42mCi.g^−1^; ICN, Basingstoke, United Kingdom), and 50μCi.ml ^−1 55^FeCl_3_ (specific activity=12.3mCi.mg^−1^, Amersham, Bucks, United Kingdom) and was gassed to pH 6.9, 95% O_2_, and 5% CO_2_. All other experimental groups utilized this solution with the following additions:

Series 1: Control solution plus 1mmol.l^−1^ ascorbic acid.

Series 2: Control solution plus 60μmol.l^−1^ iron sulfate (FeSO_4_).

Series 3: Control solution plus 12μmol.l^−1^ either cadmium sulfate (CdSO_4_), copper sulfate (CuSO_4_), manganese sulfate (MnSO_4_), or zinc sulfate (ZnSO_4_).

PCTs with 3–6 surface loops were identified by injection of a small droplet of Sudan Black-stained castor oil into randomly selected tubules. The perfusion pipette was placed downstream of the initial puncture site, and timed collections were made from the sections of the PCT isolated from the rest of the tubule by the injection of mineral oil blocks into the tubule lumen.

At the end of an experiment, arterial blood (5-10ml) was withdrawn *via* the descending aorta and the animal euthanized by administering an overdose of anesthetic. The perfused sections of tubules were filled with the silicone rubber solution, and the filled kidney removed and stored overnight in deionized water at 4°C. The length of the perfused section of tubule was determined from dissection of the silicone rubber casts as previously described (Green et al., 1974).

### Measurement of Tubular Fluid, Urine, and Blood Variables

The collected tubular fluid was dispensed under oil, and the volume was measured from the diameter of the droplet using a calibrated eyepiece micrometer. The concentration of Fe in the collected fluid was measured using electrothermal atomic absorption spectrophotometer (ETASS; Perkin Elmer Zeeman 3,030). Iron concentration was calculated by the standard addition technique (Perkin Elmer, United States). In microperfusion experiments, ^14^C-inulin and ^55^FeCl_3_ were measured by dual label liquid scintillation counting. The concentration of sodium in the perfusate and collected fluid was measured by ETASS using a previously described protocol (Shalmi et al., 1994). Iron in serum and urine was measured by flame atomic absorption spectrophotometry (FAAS; Perkin Elmer 3,100) using previously described tricarboxylic acid (TCA) precipitation protocol (Olson and Hamlin, 1969; Fernandez and Kahn, 1971; Wareing et al., 2000). To measure ultrafiltrable ion concentrations, plasma was spun at 4800g RCF for 30min through a 30kDa or 10kDa filter (Centrifree micropartition system; Amicon). Filtrate was assayed using FAAS. Fluid reabsorptive rate (J_V_, nl.mm^−1^.min^−1^) was calculated using the following equation:


$$J_{V}=V_{P}\left(1-In_{p}/In_{c}\right)/L$$

where V_p_ is the tubular perfusion rate (nl.min^−1^), In_p_ and In_c_ are the concentrations of ^14^C-inulin in perfused and collected fluids, respectively, and L is the tubule length (mm). Net sodium flux (J_Na_) was calculated using the following equation:


$$J_{Na}=V_{p}[C_{Nap}-C_{Nac}\left(In_{p}-In_{c}\right]/L$$

where J_Na_ is net sodium transport (pmol.mm^−1^.min^−1^), and C_Nap_ and C_Nac_ are concentrations of sodium (in mmol.l^−1^) in the perfusion solution and the collected fluid, respectively. To calculate the net Fe flux (J_Fe_), the specific activity of the radiolabeled Fe was used to convert the percentage ^55^Fe flux data to concentration data and these values were then substituted for sodium concentration in the standard equation (Wilson et al., 1997). A positive value for flux indicated reabsorption from the tubule lumen.

Statistical significance for microperfusion experiments was assessed using students *t* test or single-factor ANOVA using Bonferroni’s multiple comparison *post hoc* test. Values for ion and water fluxes and isotope recoveries are presented as means ± SEM throughout the text, where *n*=number of tubules unless stated otherwise.

## Results

### Whole Animal Data

Serum Fe concentration measured using the standard TCA precipitation protocol was 50.5±3.9μmol.l^−1^ (*n*=23). The MAP at the time when collections were made was 103.6±0.6mmHg (*n*=26). The Fe concentration measured in serum that had been spun through micropartition columns was 4.5±0.4μmol.^−1^ (*n*=15) for the 30kDa cutoff filter and 2.5±0.2μmol.l^−1^ (*n*=23) for the 10kDa cutoff filter. These values are consistent with our previous measurements and indicated that the serum contained an ultrafilterable component of Fe. Urine Fe concentration was 4.5±0.4μmol.l^−1^ (*n*=23).

### Tubular Fluid Collection From Proximal Convoluted Tubules

**Figure 1:**
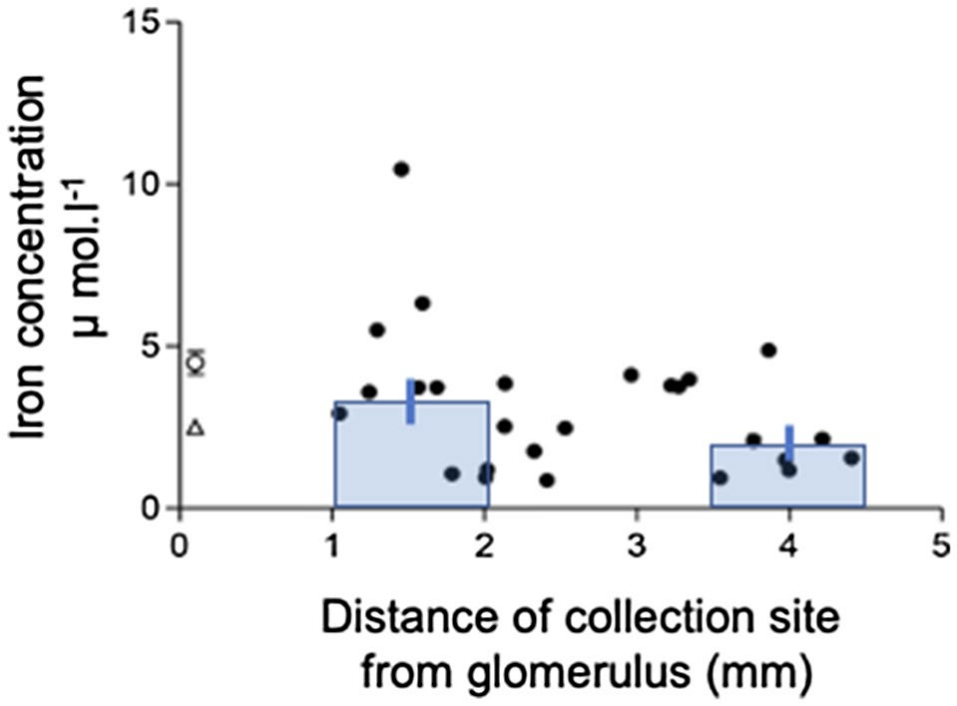
Iron concentration profile along the S2 renal proximal convoluted tubule (PCT). Iron concentration in collection measured by FAAS plotted against distance of collection from glomerulus. Data are plotted for individual tubular fluid collections from 7 animals. Equation of regression line *y*=5.026–0.738x; *n*=22; *r*^2^=0.13. Bars show mean±SEM for collections made in early part of S2 (1-2mm from glomerulus) 3.3±0.6μmol.l^−1^, *n*=10) and late part of S2 (3.5–4.5mm from glomerulus) 2.1±0.5μmol.l^−1^, *n*=7. Comparison of these two means by unpaired *t* test yielded a value of *p*=0.13.

The mean tubular fluid collection time was 19.4±0.7min (*n*=26), and the mean volume collected was 249.3±2.0nl (*n*=26). The concentration of Fe in the collected fluid ranged from 0.9 to 10.5μmol.l^−1^. The mean Fe concentration in the collected tubular fluid was 3.0±0.4μmol.l^−1^ (*n*=26) and was significantly lower than serum Fe (*p*<0.001, *n*=23). Plotting Fe concentration in collected fluid against distance of the collection site from the glomerulus gave a scatter of points (Figure 1). Least squares regression analysis yielded a regression line described by the equation *y*=5.026–0.738x; *n*=26. However, the correlation coefficient was not significant at the 5% level (*r*^2^=0.13). Pooling the concentrations of Fe measured in collections from the initial S2 segment (1–2mm from the glomerulus) and the collections made in the last S2 segment (3.5–4.5mm from glomerulus) gave means of 3.3±0.6μmol.l^−1^ and 2.1±0.5μmol.^−1^, respectively (Figure 1). Comparing these two means by unpaired *t* test yielded a *p* value of 0.13 and deemed the values to be not statistically different at the *p*<0.05 level.

### Microperfusion of Proximal Convoluted Tubules

Recovery of perfused ^14^C-inulin was not significantly different from 100% for all experimental groups, and therefore, perfusate was not lost during perfusion and differences between the amount of Fe perfused and recovered must have therefore been due to tubular events. The mean duration of collection, the mean length of the tubules perfused, and the MAP at the time of each collection were not statistically different between groups (Table 1).

**Table 1:** Tubular parameters for tubular microperfusion study.

	MABP at time of collection (mmHg)	Mean collection duration (mins)	^14^C-inulin recovery (%)	Tubule length (mm)
Control (19)	103.9 ± 1.3	3.2 ± 0.2	99.2 ± 1.2	1.6 ± 0.2
Ascorbate (15)	101.7 ± 0.6	3.0 ± 0.2	99.2 ± 1.2	1.6 ± 0.2
Iron (12)	101.1 ± 0.3	3.2 ± 0.2	100.6 ± 1.5	2.0 ± 0.3
Copper (9)	105.4 ± 1.3	2.6 ± 0.4	100.0 ± 1.7	1.5 ± 0.2
Zinc (7)	101.3 ± 0.5	3.3 ± 0.4	99.7 ± 1.8	1.8 ± 0.2
Manganese (8)	102.5 ± 1.21	3.3 ± 0.4	100.4 ± 1.7	1.4 ± 0.1
Cadmium (9)	100.9 ± 0.4	2.9 ± 0.3	97.2 ± 1.8	1.5 ± 0.1

*Values are means±SEM; number of tubules in parentheses; ^14^C-inulin recoveries were not significantly different from 100%. MABP at time of collection, collection duration, and tubule lengths were comparable in each group*.

**Figure 2:**
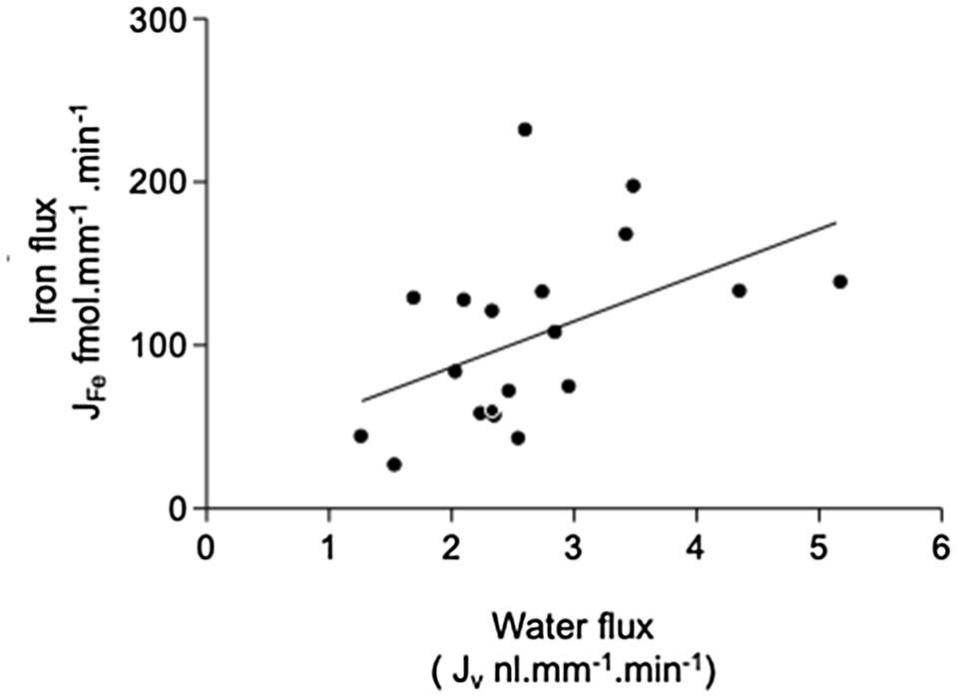
Relationship between iron and fluid reabsorption in the perfused PCT (control data only). Iron flux J_Fe_ plotted against water flux J_v_. Data are plotted for individual tubular fluid collections from 5 animals, *n*=19 collections. Equation of regression line y=28.26+29.93x; *n*=19; *r*^2^=0.23. The correlation coefficient *r*^2^=0.23 was significant at the 5% level, indicating that there was a small significant positive correlation between the reabsorption of iron and water reabsorption.

Perfusion of PCTs with a physiological solution containing 12μmol^−1 55^FeCl_3_ (control) resulted in recovery of 66.2±3.6% (*n*=19) of perfused ^55^Fe. This value was significantly different from 100%, and therefore, one-third of Fe we presented to the PCT was reabsorbed. Normalizating the amount of Fe reabsorbed per unit length of tubule gave a value of 105.2±12.7 fmol.mm^−1^.min^−1^ (n=19). Plotting J_Fe_ against J_v_ gave a scatter of points described by the line y=28.3x+29.9 (*n*=19, Figure 2). The correlation coefficient *r*^2^=0.23 was significant at the 5% level, and therefore, reabsorption of Fe was influenced by water reabsorption.

### Series 1

#### Effect of Ascorbate

**Figure 3:**
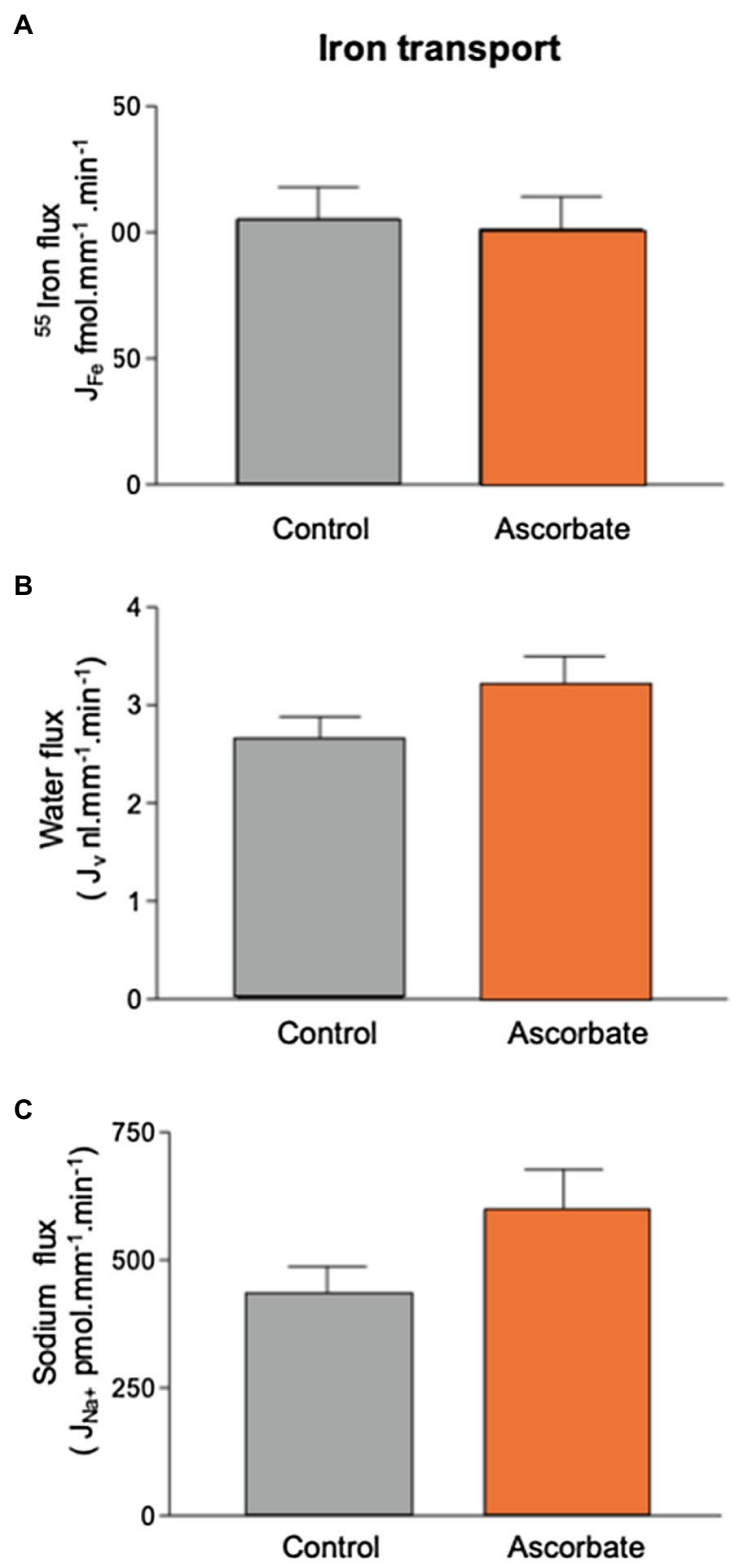
Effect of 1mmol.l^−1^ ascorbate on ^55^Fe, water, and Na^+^ flux in microperfused renal PCT. Surface S2 PCTs were microperfused *in vivo*, and the effect of 1mmol.l^−1^ ascorbic acid on ^55^Fe flux determined. Control perfusate contained 12μmol.l^−1 55^FeCl_3_. Ascorbate perfusate consisted of control perfusate plus 1mmol.l^−1^ ascorbic acid **(A)** Mean Fe flux of 105.2±12.7 fmol.mm^−1^.min^−1^ (*n*=19) was measured. Addition of 1mmol.l^−1^ ascorbic acid resulted in a mean Fe flux of 101.2±13.0 fmol.mm^−1^.min^−1^ (*n*=15). **(B)** Water flux by microperfused PCT was not significantly affected by the addition of ascorbic acid (2.7±0.2; *n*=19 to 3.2±0.3nl.mm^−1^.min^−1^; *n*=15). **(C)** Sodium flux was slightly elevated by inclusion of ascorbate, but the difference did not reach statistical significance at 0.5% level (434.9±52.2; *n*=19 to 598±79 pmol.mm^−1^.min^−1^; *n*=15).

Addition of 1mmol.l^−1^ ascorbate to the luminal perfusate did not significantly alter the transport of ^55^Fe (101.2±13.0 fmol.mm^−1^.min^−1^ (*n*=15). This treatment produced small increases in sodium reabsorption (434.9±52.2; n=19 to 598±79pmol.mm^−1^.min^−1^; *n*=15) and water transport (2.7±0.2; *n*=19 to 3.2±0.3ml.mm^−1^.min^−1^; *n*=15). These increases were not statistically significant at the 5% level (t test, *p*=0.08 and 0.16, respectively; Figure 3). Because ascorbate had no effect on PCT Fe reabsorption, it was not included in further experiments.

### Series 2

#### Effect of Fe Sulfate (FeSO_4_)

**Figure 4:**
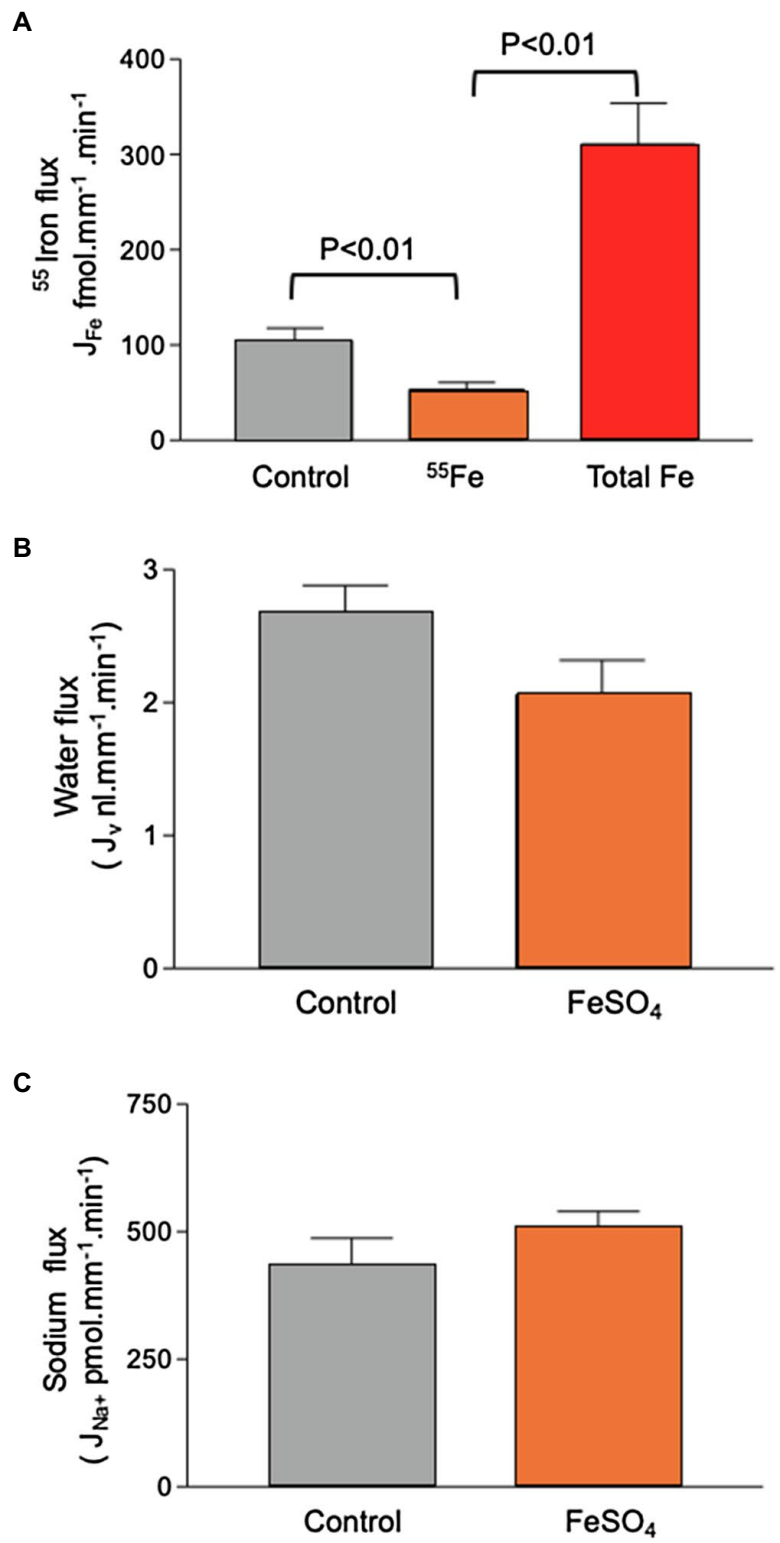
Effect of 60μmol.l^−1^ FeSO_4_ on ^55^Fe, water, and Na^+^ flux in microperfused renal PCTs. Surface S2 PCTs were microperfused *in vivo*. **(A)** Addition of 60μmol.l^−1^ FeSO_4_ reduced ^55^Fe flux from 105.2±12.7 fmol.mm^−1^.min^−1^ to 53.5±7.5 fmol.mm^−1^.min^−1^ (*n*=11; *p*<0.01). Recalculating the molar activity of ^55^Fe to take into account the addition of 60μmol.l^−1^ iron denoted “Total Fe” revealed that iron transport as a whole increased to 310.5±43.4 fmol.mm^−1^.min^−1^(*n*=11; p<0.01). **(B)** Mean water flux was reduced by inclusion of 60μmol.l^−1^ FeSO_4_ from 2.7±0.2 to 2.1±0.3nl.mm^−1^.min^−1^ (*n*=11), but the observed reduction was not statistically significant at p,0.5 level. **(C)** Sodium flux by microperfused PCT (428.6±57.2; *n*=19. Addition of 60μmol.l^−1^ FeSO_4_ gave a mean value of 514.80±28.6nmol.mm^−1^.min^−1^; *n*=11).

Addition of 60μmol.l^−1^ FeSO_4_ to the luminal perfusate did not affect sodium reabsorption and caused a small, but not statistically significant, decrease in water reabsorption from 2.7±0.2 to 2.1±0.3nl.mm^−1^.min^−1^ (*n*=11, Figure 4). Transport of ^55^Fe was reduced from 105.2±12.7 fmol.mm^−1^.min^−1^ to 53.5±7.5 fmol.mm^−1^.min^−1^ (*n*=11; *p*<0.01). Recalculating the molar activity of ^55^Fe to take into account the addition of 60μmol.l^−1^ Fe revealed that Fe transport as a whole increased to 310.5±43.4 fmol.mm^−1^.min^−1^(*n*=11; *p*<0.01). This showed that the control perfusion of 12μmol^−1^ Fe, although three times higher than the concentration of Fe we measured in the PCT collections, did not saturate the transport pathway.

### Series 3

#### Effect of Copper (CuSO_4_), Zinc (ZnSO_4_), Manganese (MnSO_4_), or Cadmium Sulfate (CdSO_4_)

**Figure 5:**
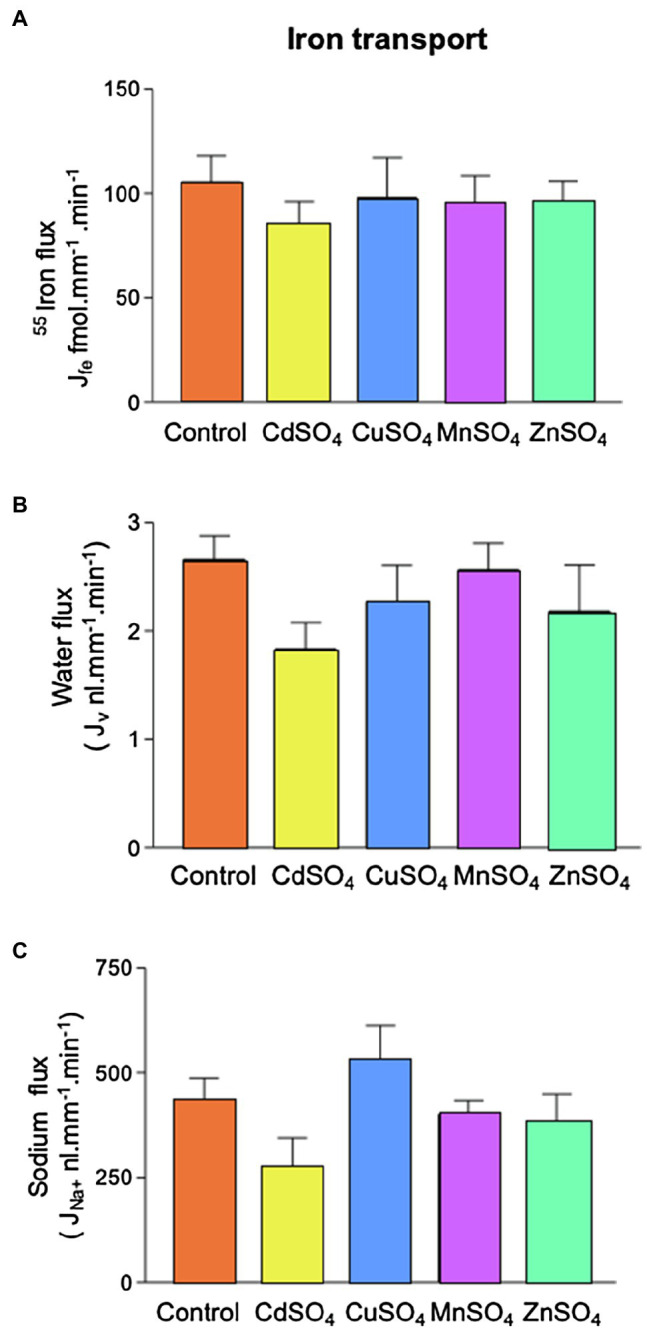
Effect of competing divalent metal addition on ^55^Fe, water and Na^+^ flux in microperfused renal PCTs. Tubules were perfused with 12μmol.l^−1 55^FeCl_3_ (control) and CdSO_4_, CuSO_4_, MnSO_4_, or ZnSO_4_ were included (12μmol.l^−1^). **(A)** ^55^Fe flux was unaffected by competing metals. **(B)** Inclusion of competing metals had no statistically significant effect on water flux. **(C)** Inclusion of competing metals had no statistically significant effect on Na^+^ flux. Number of replicates were as follows: control =19, CdSO_4_=9, CuSO_4_=9 MnSO_4_=8, or ZnSO_4_=7.

Addition of equimolar CuSO_4_, MnSO_4,_ or ZnSO_4_ (12μmol.l^−1^) had no significant effect on Fe reabsorption (Figure 5). This suggested that neither copper, manganese, nor zinc competes with Fe for transport. CdSO_4_ caused marginal decreases in water and sodium reabsorption (*p*=0.25 and 0.15, respectively; ANOVA), but Fe absorption was unaffected by these reductions in transport.

## Discussion

The aim of the current study was to determine the Fe concentration in ultrafiltrate collected from the lumen of rodent S2 proximal tubule and to determine whether Fe is reabsorbed by the PCT. To do this, we utilized *in vivo* renal micropuncture and measured Fe concentration in samples of ultrafiltrate collected from S2 nephron segment. We also performed divalent metal competition studies to begin to functionally characterize the Fe reabsorptive pathway.

Tubular fluid collected from rat PCT contained Fe. This finding suggests Fe is either filtered by the glomerulus or secreted into the tubular lumen. By comparing tubular Fe concentration to the distance, a collection was made from the glomerulus and we determined whether Fe was reabsorbed or secreted. Because 50–60% of the water in the ultrafiltrate is reabsorbed from the PCT, a solute that is not reabsorbed or one that is secreted would increase in concentration the further a collection was made from the glomerulus (Rector, 1983). In the case of isosmotic reabsorption, no change in concentration would be expected and for higher rates of reabsorption the concentration of an ion/solute along the PCT would decrease. By comparing collections made in the first and last accessible millimeter of the S2 PCT, it was very evident that there was no discernable increase in PCT Fe and the concentration remained relatively constant or if anything decreased slightly along the PCT. These findings when taken together strongly suggest Fe is not secreted into the tubule lumen, but that it is filtered by the glomerulus and reabsorbed along the proximal tubule.

Comparing Fe flux and water flux enabled us to determine whether reabsorption of Fe might be linked to water movement. We found a correlation between water and Fe flux, indicating that the two processes were linked. This was not unexpected given that movement of a solute across a membrane generates an osmotic gradient. On the other hand, movement of water out of the tubule serves to increase the effective concentration of a solute and hence increases the solute concentration gradient thus favoring transport out of the tubular lumen. It was not possible from our experiments to definitively determine which mechanism led to the observed coupled movement; however, the finding that PCT Fe and water movement were linked reinforces the conclusion that Fe is reabsorbed from the PCT.

To begin to functionally characterize the proposed reabsorptive pathway, we performed a series of PCT microperfusion experiments. We perfused PCTs and measured the flux of ^55^Fe out of the proximal tubule as a marker for Fe reabsorption. To ensure that the PCT was functioning normally, we simultaneously measured sodium and water flux and obtained values very comparable to those previously reported by our laboratory under similar perfusion conditions (Wareing and Green, 1994; Wareing et al., 1995). The mean recovery of Fe from PCT’s perfused with a solution containing 12μmol.l^−1^ Fe was 66%. Therefore, on average, a third of the Fe presented to the PCT was taken up by the tubule. The rate of Fe transport measured was 105.2±12.7 fmol. mm^−1^.min^−1^, and since this is the first measurement of PCT Fe flux, it is not possible to compare it to other published data, and while the fluxes of other ions out of the PCT (e.g., Na, Ca, K, or Mg) have been measured by our group and others groups given the methodological differences, it is not possible to directly compare these values.

Adding unlabeled Fe to the perfusate decreased the apparent unidirectional ^55^Fe flux. This was due to unlabeled Fe competing with ^55^Fe, possibly for a fixed number of transporter carrier binding sites. Recalculating the molar activity of ^55^Fe to take into account the addition of unlabeled Fe revealed that increasing the Fe concentration five-fold caused a three-fold increase in Fe flux. This outcome is important for two reasons: Firstly, it shows that our experimental protocol was capable of uncovering decreased or increased Fe flux; secondly, it showed that the transport pathway function was concentration dependent.

The membrane-bound metal transporter proteins DMT1, ZIP8, and ZIP14 are expressed in the mammalian PCT (Wang et al., 2012; Van Raaij et al., 2018). These proteins are selective for several divalent metals including Fe^2+^. With this in mind, our next aim was to determine the characteristics of PCT Fe flux. Firstly, we looked at the effect of including ascorbate in the perfusate, reasoning that DMT1, ZIP8, and ZIP14 mediate the transport of Fe^2+^ and the perfusate we used contained predominately Fe^3+^; thus, inclusion of ascorbate would be expected to increase Fe flux (Sparkman et al., 2011; Illing et al., 2012; Wang et al., 2012). We found ascorbate did not alter Fe flux suggesting that ascorbate was not necessary for PCT Fe translocation. Ferrous Fe^2+^ is promoted by inclusion of ascorbic acid; therefore, the finding that ascorbate did not increase flux argues against involvement of DMT1, ZIP8, or ZIP14 in transporting Fe out of the PCT. Interestingly, ascorbate caused water flux to increase marginally. This might reflect the transport activity of ascorbate by the Na+−coupled L-ascorbic acid transporter SVCT1. SVCT1 is expressed in the PCT, and like SGLT1 is likely to transport water and its cognate substrate (Castro et al., 2008; Mackenzie et al., 2008; Bürzle et al., 2013).

To further probe the reabsorptive pathway, we performed ion competition experiments. A prominent characteristic of DMT1, ZIP8, and ZIP14 is that in addition to Fe, these proteins transport other divalent metals: DMT1 has substrate selectivity for Cd^2+^, Co^2+^, Mn^2+^, and to a lesser extent Zn^2+;^ ZIP 8 has selectivity for Cd^2+^, Co^2+^, Zn^2+^, and to a lesser extent Mn^2+^ and ZIP 14 has the capacity to mediate transport of Cd^2+^, Mn^2+^, and Zn^2+^(Sparkman et al., 2011; Illing et al., 2012; Wang et al., 2012). In light of this, we microperfused tubules with solutions containing Cd, Zn, Cu, or Mn. Addition of competing metals did not significantly alter Fe, sodium, or water flux, although addition of CdSO_4_ to the perfusate caused a marginal decrease in sodium and water flux that did not reach statistical significance. Taken together, these data suggest DMT1, ZIP8, and ZIP14 are unlikely major contributors to unidirectional Fe flux out of the PCT.

In our previous work, we proposed that DMT1 is responsible for transport of Fe across the apical membrane of the late loop of Henle and early distal tubule because we showed DMT1 is expressed in the apical plasma membrane of these segments and transport of Fe was reduced by inclusion of other divalent metals which are known ligands of DMT (Wareing et al., 2000; Ferguson et al., 2001). In contrast, DMT1 is unlikely to transport Fe across the apical PCT membrane based on the functional studies presented in this work and this is supported by robust immunolabelling studies showing DMT1 expressed in late endosomes and lysosomes of PCT cells in rodents and not in the apical plasma membrane (Abouhamed et al., 2006).

If not DMT1, then could ZIP8 or ZIP14 be responsible for PCT Fe flux? In order to fulfil this role, these transporters would have to be expressed in the apical PCT membrane. Unfortunately, to date, it is not possible to discern with any degree of certainty whether these proteins are expressed in this membrane or like DMT1 whether they are expressed in a subcellular compartment. Furthermore, in the current study none of the competing divalent metals we trialed which are known ligands of ZIP8 or ZIP14 inhibited Fe transport indicating that in the absence of pathology, neither of these proteins mediate transport of Fe in the segments we investigated (mainly S2). In support of this tentative conclusion, ZIP8 has negligible transport activity at pH 6.5, and the prevailing pH in the S2 segment is ~6.7 (Dubose et al., 1979; Wang et al., 2012). In comparison, ZIP14 shows some activity at pH6.5 (Sparkman et al., 2011); however, the lack of effect of zinc on Fe transport argues strongly against the proposed involvement of this protein in PCT apical membrane Fe flux (Liuzzi et al., 2006; Fujishiro et al., 2012).

Other potential candidate mechanisms are apparent in the PCT. The megalin/cubilin receptor complex and TFR1 are both expressed in the apical PCT membrane are likely to be responsible for reabsorbing filtered transferrin bound Fe (Christensen et al., 2012; Smith et al., 2019). Our micropuncture experiments simply demonstrated that Fe was present in ultrafiltrate and that the Fe concentration profile along the proximal tubule strongly indicated reabsorption; however, we did not assess what species of Fe was present. The tubular perfusion experiments we performed used a perfusate which was protein-free and contained Fe in its unbound state – sometimes referred to as non-transferrin bound Fe. We specifically designed the experiments to enable us to functionally test for the presence of DMT1, ZIP8, and ZIP14 since these transporters translocate unbound Fe. Clearly, future experiments should aim to examine the role of receptor-mediated mechanisms while bearing in mind the observations of the current study that indicate the presence of transport of elemental unbound- Fe.

**Figure 6:**
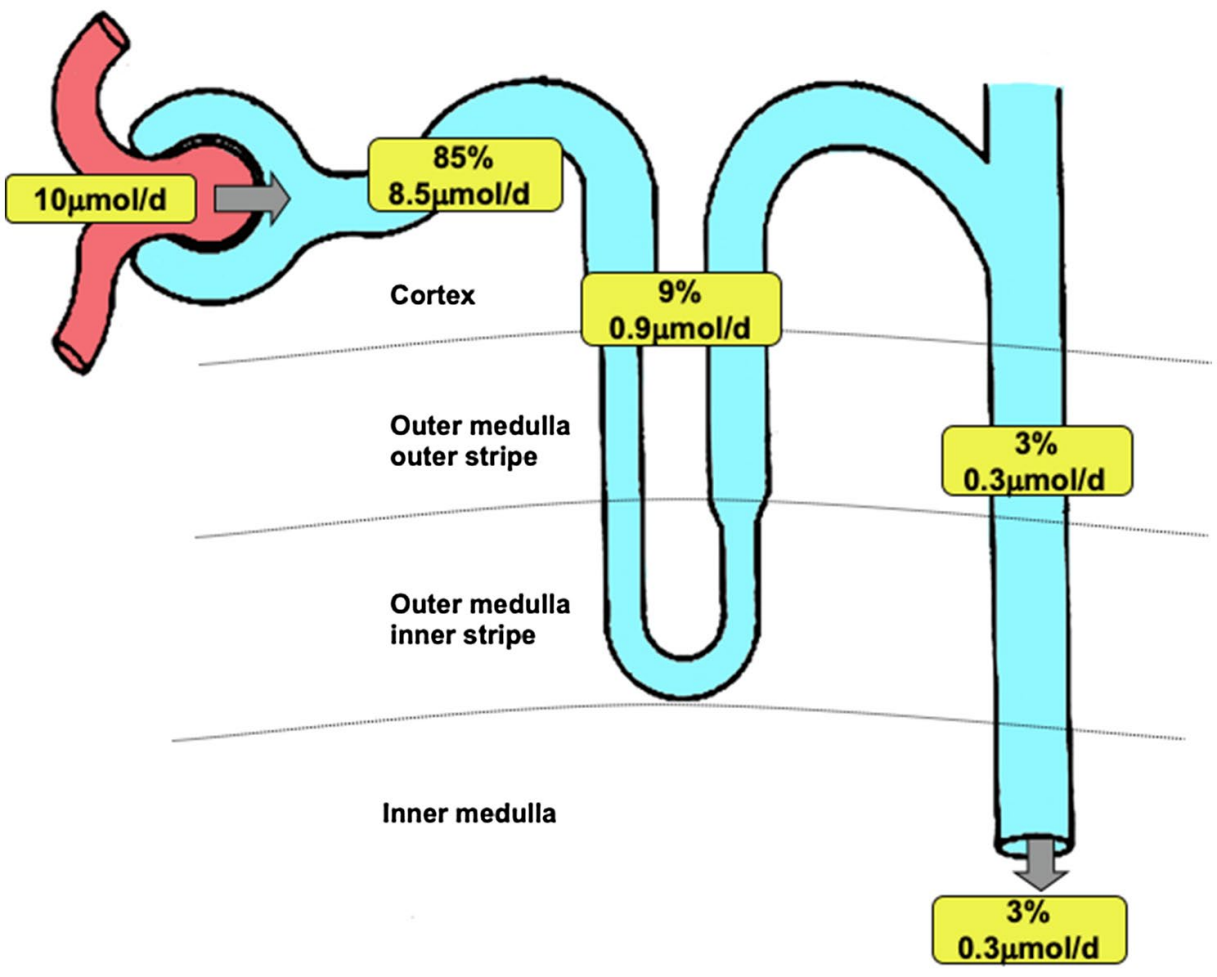
Renal iron transport. Model of renal iron handling along the nephron showing estimated quantitative contribution of three groups of nephron segments. Percentage data refer to the amount of filtered iron reabsorbed. Values apply to a 250g Wistar rat and are based on data presented in this manuscript and Wareing et al., 2000). Filtered load of iron was calculated assuming a glomerular filtration rate of 2.25l.day^−1^ (Bijsterbosch et al., 1981) and taking the 30kDa ultrafiltrate iron concentration of 4.48μmol.l^−1^ as representative of the concentration of iron filtered. Iron remaining at the end of the PCT was calculated assuming 60% of filtered water is reabsorbed along PCT and taking the end PCT iron concentration to be 1.8μmol.l^−1^ (from Figure 1). PCT reabsorption of iron was calculated by subtracting end PCT iron concentration from the filtered load of iron. The amount of iron reabsorbed by the loop of Henle or DCT and collecting ducts was calculated using the urinary iron concentration as a starting point (value taken Wareing et al., 2000,) and assuming 20% of injected iron was reabsorbed downstream of the PCT (value taken Wareing et al., 2000). Iron present at the start of the DCT was calculated assuming 50% of injected iron was reabsorbed downstream of the DCT. Iron reabsorbed by loop of Henle was calculated by subtracting the calculated value for the DCT from the measured iron concentration at the end of the PCT (from Figure 1).

Overall based on the data presented in this work, what is the contribution of renal Fe reabsorption to Fe balance? We propose that in addition to its role as a source of EPO, the kidney is critical for maintaining Fe balance due to the following reasoning: Within serum, the concentration of Fe measured using the TCA precipitation method was 50.5±3.9μmol.l^−1^. The majority of this represents Fe bound to transferrin, and some of this passes the glomerular filtration barrier and becomes a component of the ultrafiltrate. The plasma volume of a 250g rat is 9.8 mls (Bijsterbosch et al., 1981); therefore, the total amount of Fe in plasma is 0.49μmol. *In vitro* we measured the ultrafiltered concentration of Fe to be 4.5μmol.l^−1^ and *in vivo* the mean PCT concentration in the S2 segment to be 3.0μmol.l^−1^. Assuming a mean glomerular filtration rate of a 250g rat is 2.25l.day^−1^, then between 6.9 and 10.1μmol Fe would be filtered per day. In terms of maintaining Fe balance, the implication of this estimate is that the total amount of Fe in plasma would be lost in less than two hours if the kidney did not reabsorb filtered Fe. From the available data, we constructed a model of renal Fe handling (see Figure 6). It can be seen that the bulk of Fe is reabsorbed in the PCT, by a non-DMT1-dependent pathway that is not shared by copper, manganese, or zinc. There is significant DMT1-mediated transport of Fe by the loop of Henle and the collecting duct system which in comparison may also mediate transport of copper and manganese and to some extent zinc.

Micropuncture is not possible in human subjects, and imaging methods are not sufficiently refined to resolve nephron distribution of Fe; however, recent data suggest that transporter proteins necessary to affect epithelial Fe transport are present in the human kidney and may in some cases be implicated in deleterious renal Fe accumulation (Van Raaij et al., 2018). And although it can be inferred that Fe is filtered by human kidneys, definitive localization studies to pinpoint the nephron expression of transporter proteins are lacking and should ideally be the focus of future studies.

In conclusion, we have demonstrated that Fe is present in ultrafiltrate in the S2 PCT. The Fe concentration profile along the PCT is indicative of PCT Fe reabsorption. The mechanism responsible for the observed flux remains obscure; however, DMT1, ZIP8, or ZIP14 are unlikely to majorly contribute. We propose that the rat kidney filters and reabsorbs Fe and this process is potentially of critical importance to Fe homeostasis.

## Data Availability Statement

The original contributions presented in the study are included in the article/supplementary material, and further inquiries can be directed to the corresponding author.

## Author Contributions

All authors listed have made a substantial, direct and intellectual contribution to the work, and approved it for publication.

## Funding

We gratefully acknowledge the financial support of the Wellcome Trust Grant no. 043322/Z/94 (CPS) and the Royal Society (CPS).

## Conflict of Interest

The authors declare that the research was conducted in the absence of any commercial or financial relationships that could be construed as a potential conflict of interest.

## Publisher’s Note

All claims expressed in this article are solely those of the authors and do not necessarily represent those of their affiliated organizations, or those of the publisher, the editors and the reviewers. Any product that may be evaluated in this article, or claim that may be made by its manufacturer, is not guaranteed or endorsed by the publisher.
